# Editorial: Axon neurobiology: updates in functional and structural dynamics

**DOI:** 10.3389/fncel.2026.1838278

**Published:** 2026-04-17

**Authors:** Haruyuki Kamiya, Dominique Debanne

**Affiliations:** 1 Department of Neurobiology, Graduate School of Medicine, Hokkaido University, Sapporo, Japan; 2 Adhesion and Inflammation, INSERM, Aix-Marseille University, CNRS, Marseille, France

**Keywords:** action potential, axon, axon initial segment, excitability, glia, node of Ranvier, potassium channel, sodium channel

Axons are essential for the long-distance propagation of action potentials across neural networks. Traditionally, axonal signaling was considered a faithful, all-or-none process (Debanne et al., 2011). However, recent studies have fundamentally revised this view (Debanne et al., 2013; Ohura and Kamiya, 2016), demonstrating that axons exhibit pronounced functional and structural tuning depending on neuronal activity (Ohura and Kamiya, 2018), developmental stage, and microenvironmental conditions (Debanne and Rama, 2011; Sasaki et al., 2011). These dynamic properties allow axons to actively shape synaptic transmission and neural circuit integration rather than simply relay electrical signals. Advances in imaging, molecular biology, and electrophysiology have been crucial to revealing these forms of axonal plasticity. This Research Topic highlights recent progress in axon neurobiology, focusing on functional and structural dynamics under physiological and pathological conditions.

Neuronal communication relies on axonal polarity and specialized excitable domains, particularly the axon initial segment (AIS) and nodes of Ranvier. Several studies in this Research Topic address how the regulation of these domains modulates axonal excitability. In myelinated axons, saltatory conduction depends on the precise clustering of ion channels at nodes of Ranvier. Using high-speed fluorescence imaging in cortical layer five pyramidal neurons, Kotler et al. characterized action potential-evoked Na^+^ and Ca^2+^ signals at nodes. Both signals were spatially restricted to nodal regions. Na^+^ transients peaked rapidly during the action potential upstroke and decayed by diffusion into internodes, with kinetics dependent on nodal length. In contrast, Ca^2+^ transients peaked later, decayed via active clearance, and recovered rapidly in a temperature-independent manner, suggesting minimal channel inactivation during single spikes.

The molecular basis of axonal excitability is further examined by Escobedo and Rasband, who reviewed potassium channel clustering at the AIS and nodes of Ranvier. At the AIS, voltage-gated Kv channels, such as Kv7 and Kv1, regulate the action potential threshold and repolarization. At nodes, recent evidence indicates that two-pore domain K2P channels, particularly TRAAK and TREK-1, play a dominant role in membrane repolarization. Precise localization of these channels is mediated by interactions with scaffolding proteins, and disruption of Kv or K2P channel organization is associated with epilepsy and neurodevelopmental disorders.

Beyond neuron-intrinsic mechanisms, glial cells are emerging as important regulators of axonal excitable domains. Garrido summarized the evidence that astrocytes and microglia interact with the AIS and nodes of Ranvier to support neuronal excitability, structural plasticity, and homeostasis. These interactions involve the regulation of the local ionic environment and secretion of signaling factors. Disruption of glia-axon interactions by injury or disease can significantly impair central nervous system function, highlighting their relevance in axonal pathology.

Dynamic modulation of action potential propagation is further illustrated by Furukawa and Kawaguchi, who demonstrated that β-adrenergic receptor activation reduces action potential amplitude and conduction velocity in cerebellar Purkinje cell axons via cAMP-dependent suppression of Na^+^ currents, with effects dependent on axonal length. Despite reduced axonal excitability, β-adrenergic signaling increased presynaptic release probability, revealing a complex relationship between axonal conduction and synaptic output.

Finally, Egawa et al. address axonal structural refinement during development. Using single-axon tracing in the chick ciliary ganglion, they showed that activity-dependent presynaptic mechanisms drive the maturation of calyx-type terminals and axonal branch pruning, resulting in one-to-one connectivity. Silencing presynaptic activity delayed terminal maturation, indicating that presynaptic activity restricts the number of post-synaptic targets innervated by each axon.

Together, these studies emphasize that axons are dynamic structures whose functional and structural plasticity are central to neural circuit operation and dysfunction. By integrating cutting-edge electrophysiology, molecular biology, and imaging approaches, this Research Topic advances our understanding of how axonal dynamics shape brain function in health and disease.
